# Correlation between of small dense low‐density lipoprotein cholesterol with acute cerebral infarction and carotid atherosclerotic plaque stability

**DOI:** 10.1002/jcla.22891

**Published:** 2019-04-06

**Authors:** Xue QiaoZhen, Meng AiGuo, Wang Tong, Li JingJing, Liu HaiYing

**Affiliations:** ^1^ Department of Clinical Laboratory Affiliated Hospital of North China University of Science and Technology Tangshan China; ^2^ Tangshan Key Laboratory of Medical Molecular Testing and Diagnosis Tangshan China

**Keywords:** acute cerebral infarction, small dense low‐density lipoprotein cholesterol, unstable plaque

## Abstract

**Background:**

Acute cerebral infarction (ACI) is seriously harmful to human health worldwide. However, at present, the risk of disease onset is still not accurately predicted for some people.

**Methods:**

Five hundred and nineteen patients with ACI and 300 healthy controls were included in this study. We divided the patients into three groups according to the results of cervical artery contrast‐enhanced ultrasound. Ninety‐five patients were in the CAS without plaque group, 108 patients were in the stable plaque group, and 316 patients were in the unstable plaque group. TC, TG, HDL‐C, LDL‐C, and sdLDL‐C were measured in all subjects.

**Results:**

The level of small dense low‐density lipoprotein cholesterol (sdLDL‐C) in the ACI group was significantly higher than that in the control group (*P* < 0.001). Logistic regression analysis showed that sdLDL‐C was an independent risk factor for ACI (OR = 1.067, 95% CI: 1.041‐1.093, *P* < 0.001); serum sdLDL‐C was significantly higher in the unstable plaque group than in the stable plaque group and plaque‐free group (*P* < 0.05, *P* < 0.001); serum sdLDL‐C was also higher in the stable plaque group than the plaque‐free group (*P* < 0.001). Logistic regression analysis showed that sdLDL‐C was an independent risk factor for unstable carotid plaques (OR = 1.053, 95% CI: 1.038‐1.068, *P* < 0.001); Spearman correlation analysis showed that sdLDL‐C test results were positively correlated with carotid plaque stability (*r* = 0.363, *P* < 0.001).

**Conclusion:**

Small dense low‐density lipoprotein cholesterol is an independent risk factor for the onset of ACI and may be an early serum marker for this disease.

## 1 INTRODUCTION

Acute cerebral infarction (ACI) is an ischemic cerebrovascular disease that is seriously harmful to human health worldwide. Its prevalence and disability rate have been increasing in recent decades.^1^ Carotid atherosclerosis (CAS) is currently considered the pathological basis for the development of ACI,^2^ in which unstable atherosclerotic plaques play an important role.^3^ Some factors, such as low‐density lipoprotein cholesterol (LDL‐C), obesity, diabetes, hypertension, sex, and age, are considered traditional risk factors for atherosclerosis; LDL‐C is considered one of the most important risk factors. However, increasing clinical results have found that these traditional risk factors can only partially predict the risk of ACI, and the risk of morbidity in a considerable number of people cannot be accurately predicted.

Low‐density lipoprotein cholesterol is heterogeneous and can be divided into high cholesterol content, large particle size (peak diameter >25.8 nm) LDL‐C A; low cholesterol content, small particle size (diameter peak <25.8 nm) LDL‐C B; and small dense low‐density lipoprotein cholesterol (sdLDL‐C).^4^ Recent studies have confirmed that sdLDL‐C has stronger atherosclerosis ability than LDL‐C and has been included in the recently reported important cardiovascular and cerebrovascular disease risk factors by the American Cholesterol Education Program Adult Treatment Group.^5^ sdLDL‐C is associated with the number of atherosclerotic plaques and with carotid stenosis caused by atherosclerotic plaque.^6^, ^7^ Studies by Alberto Zambon^8^ have suggested that sdLDL‐C has a significant effect on carotid plaque cell composition.

Despite the significant role of sdLDL‐C in atherosclerosis,^6^ whether this relation is consistent with the existing research conclusions, whether sdLDL‐C is related to the stability of CAS plaque, and whether sdLDL‐C can better predict the risk of ACI have been under studied.

Therefore, this study evaluated patients diagnosed with ACI in our hospital and measured the serum sdLDL‐C level. The aims were as follows: (a) to observe the relationship between serum sdLDL‐C level and ACI by observing the level of sdLDL‐C in patients with normal LDL‐C levels. (b) To determine whether serum sdLDL‐C can be used as a serum marker for predicting ACI by verifying whether there is a correlation between serum sdLDL‐C levels and CAS plaques with different levels of stability.

## 2 MATERIALS AND METHODS

### 2.1 Patient population and protocol

From November 2017 to November 2018 in the Department of Neurology at the Affiliated Hospital of North China University of Technology, 519 patients with ACI, including 342 males and 177 females, with an average age of 60.62 ± 11.04 years were recruited. We divided the patients into three groups: 95 patients were allocated to the CAS without plaque group, 108 patients were allocated to the stable plaque group, and 316 patients were allocated to the unstable plaque group. Participant inclusion was determined based on the following: (a) The diagnosis is in accordance with the diagnostic criteria established by the Fourth National Conference on Cerebrovascular Diseases, (b) the diagnosis was confirmed by cranial CT or MRI, and (c) the first cerebral infarction was within 72 hours. Participant exclusion was determined based on the following: (a) patients with a previous history of cerebral infarction, (b) patients with severe cardiovascular disease, such as coronary heart disease and myocardial infarction, (c) patients with metabolic disease, such as diabetes, and (d) patients who have been taking lipid‐lowering drugs in the past two months. The patients’ clinical parameters included age, sex, smoking habit, alcohol consumption, the presence of hypertension or diabetes, and body mass index (BMI). Patients who smoked >1 cigarettes per day for over one year were considered smokers, and patients who consumed at least two alcoholic drinks per day for over 1 year were considered drinkers. The diagnostic criteria for hypertension are based on the diagnostic and classification criteria from the 2010 Chinese Hypertension Guidelines. Diabetes was diagnosed using current WHO Diabetes Diagnostic Guidelines. Hyperlipidemia was diagnosed based on the 2007 Guidelines for the Prevention and Treatment of Abnormal Blood Lipids in Chinese Adults. In the control group, 300 healthy physical examinations were selected, including 199 males to 101 females, and their average age was 59.33 ± 10.46 years.

### 2.2 Intravascular ultrasound imaging and analysis

Carotid ultrasound examination method: Siemens Acuson S2000 color Doppler ultrasound diagnostic instrument with 914 probe frequency of 4.0~9.0 MHz. The subject was placed in the supine position. The carotid artery was cut at 2 cm above the carotid sinus level and at 1.5 cm from the carotid sinus level, and a longitudinal incision was performed. The arterial bifurcation, internal carotid artery, and external carotid artery were scanned, and the carotid intima‐media thickness (IMT) was measured. Plaques are focal structures of at least 0.5 mm or 50% of the surrounding IMT value that are encroaching into the arterial lumen or focal structures that demonstrate a thickness >1.5 mm as measured from the intima‐lumen interface to the media‐adventitia interface; otherwise, structures were not considered as plaques.^9^ Contrast‐enhanced ultrasound (CEUS) examination method: After the target plaque is found by standard ultrasound examination, the image is adjusted and partially magnified to clearly show the plaque morphology while the patient is calmly breathing. The ultrasound contrast agent is produced by Bracco International BV. Sonovi (injection sulfur hexafluoride microbubble 59 mg lyophilized powder) dissolved in 5 mL of physiological saline (0.9%) and shaken well (microbubble concentration 5 mg/mL); 2 mL of contrast agent was initially administered in the median vein of the elbow, and then, 5 mL of physiological saline (0.9%) was injected. The timing of contrast injection was preplanned and noted, and the image was collected. Contrast‐enhanced neovascularization was evaluated as follows: grade 0: no enhancement in the plaque; grade 1: contrast agent microbubbles appear at only the base or middle of the plaque along the direction of the plaque thickness; and grade 2: contrast agent microbubbles appear near the intima along the direction of plaque thickness.^10^ The stability of the plaque is defined by the extent of neovascularization in the plaque and the extent of plaque‐induced stenosis as suggested by the carotid ultrasound contrast.^11^ Neovascularization in the plaque with a grade of 0‐1 and stenosis of <50% indicate a stable plaque; neovascularization in the plaque with a grade of 2 or stenosis of 50%‐99% indicates an unstable plaque.^12^

### 2.3 Blood sampling and measurement of lipids

A total of 5 mL of venous blood were collected after fasting (>12 hours) to test the total cholesterol (TC), triglyceride (TG), LDL‐C, high‐density lipoprotein cholesterol (HDL‐C), and sdLDL‐C levels. Venous blood was collected using a procoagulant blood collection tube with an inert separation gel at the bottom (Liuyang Sanli Medical Technology Development, Inc). After blood collection, the tube was quickly inverted and mixed. After standing for a period of time, the serum was separated by centrifugation at 1700 *g* for 10 minutes and immediately detected on the machine. The above items were tested using a Beckman Coulter AU5800 machine (Beckman Coulter, Inc, Brea, CA). sdLDL‐C was detected by the peroxidase method, and the reagents were all from Beijing Jiuqiang Biotechnologies, Inc Quality control was performed using the company's quality control products.

### 2.4 Statistical analysis

Statistical analysis was performed using IBM SPSS statistical software version 17.0 (SPSS Inc, Chicago, IL. A 2‐sided *P*‐value of 0.05 was considered significant. The Kolmogorov‐Smirnov test was selected to assess the normality of the calculated parameters. Measurement data are shown as the mean ± standard deviation (SD). Student's *t* test was used for comparison between the two groups, one‐way ANOVA was used for comparison between multiple sample means, and pairwise comparisons were performed using Bonferroni's *t* test. The chi‐squared test was used for categorical variables. Independent risk factor analysis was performed using logistic regression. Correlation analysis between sdLDL‐C and TG and between sdLDL‐C and CAS stability in ACI patients was performed by Pearson correlation analysis, Spearman correlation analysis, and partial correlation analysis. The diagnostic efficacy of unstable plaques in ACI patients with CAS was verified using the receiver operating characteristic (ROC) curve.

## 3 RESULTS

### 3.1 Comparison of baseline data between the ACI group and the control group

A total of 519 patients in the ACI group and 300 age‐ and sex‐matched patients were selected. The proportion of patients with hypertension and hyperlipidemia and the TC, TG, LDL‐C, HDL‐C, and sdLDL‐C levels in the ACI group were significantly different from those in the control group (*P* < 0.01, Table 1).

**Table 1:** Comparison of three sets of baseline data

	ACI (n = 519)	Control (n = 300)	*t/χ^2^*	*P‐*value
Age (y), $\overset{-}{x}$ * ± s*	60.62 ± 11.04	59.33 ± 10.46	*t* = 1.645	0.100
Male, n (%)	342 (65.9)	199 (66.3)	*χ^2^*=0.016	0.899
BMI (kg/m^2^), $\overset{-}{x}$ * ± s*	26.90 ± 4.64	26.57 ± 4.57	*t* = 0.035	0.972
Smoking, n (%)	211 (40.7)	117 (39)	*χ^2 ^*= 0.217	0.641
Drinking, n (%)	200 (38.5)	100 (33.3)	*χ^2 ^*= 2.217	0.137
Diabetes mellitus, n (%)	97 (18.7)	58 (19.3)	*χ^2 ^*= 0.051	0.821
Hypertension, n (%)	323 (62.2)	101 (33.7)	*χ^2 ^*= 62.141	0.000
Hyperlipidemia, n (%)	336 (64.7)	88 (29.3)	*χ^2 ^*= 95.450	0.000
TC (mmol/L), $\overset{-}{x}$ * ± s*	5.19 ± 1.06	4.85 ± 0.89	*t* = 4.578	0.000
TG (mmol/L), $\overset{-}{x}$ * ± s*	1.81 ± 1.06	1.42 ± 0.83	*t* = 5.499	0.000
LDL‐C (mmol/L), $\overset{-}{x}$ * ± s*	3.38 ± 0.92	3.09 ± 0.80	*t* = 4.521	0.000
HDL‐C (mmol/L), $\overset{-}{x}$ * ± s*	1.32 ± 0.34	1.44 ± 0.31	*t *= −4.914	0.000
sdLDL‐C (mg/dL), $\overset{-}{x}$ * ± s*	38.61 ± 18.75	23.98 ± 9.96	*t* = 14.574	0.000

### 3.2 Correlation analysis between sdLDL‐C and TG

**Figure 1:**
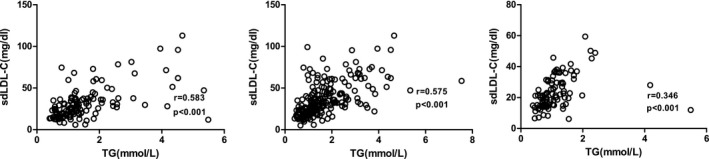
Correlation between sdLDL‐C and TG

Pearson correlation analysis and partial correlation analysis were used to evaluate the correlation between sdLDL‐C and TG and between TC and LDL‐C. It was found that sdLDL‐C was positively correlated with TG (*P < *0.001), as shown in Table 2 and Figure 1.

**Table 2:** Correlation coefficient between sdLDL‐C and TG

	All subjects	All subjects (after controlling for the influence of TC and LDL‐C)	ACI	Control
TG	0.583	0.490	0.575	0.346

### 3.3 High sdLDL‐C/LDL‐C predicts onset of ACI even with a normal LDL‐C level

The sdLDL‐C level was higher in the high LDL‐C group of patients with ACI than in the normal LDL‐C group (*P < *0.05) (Table 3).

**Table 3:** Comparison of sdLDL‐C between the normal LDL‐C group (<2.59 mmol/ L) and high LDL‐C group of patients with ACI

	Normal LDL‐C (n = 83)	High LDL‐C (n = 436)	*t‐*value	*P‐*value
sdLDL‐C (mg/dL), $\overset{-}{x}$ *± s*	34.53 ± 15.87	41.55 ± 18.41	5.896	0.038

There was no significant difference in sex, age, alcohol use, smoking status, and TC, TG, LDL‐C, and HDL‐C level between the ACI group with normal LDL‐C levels and the control group (*P* > 0.05). There was a statistically significant difference in the ratio of sdLDL‐C to LDL‐C and the level of serum sdLDL‐C between the ACI group with normal LDL‐C levels and the control group (*P < *0.05, Table 4).

**Table 4:** Comparison of serum sdLDL‐C level between the ACI group and control group with normal LDL‐C levels (<2.59 mmol/ L)

	ACI (n = 83)	Control (n = 71)	*t/χ^2^*	*P‐value*
Age (y), $\overset{-}{x}$ * ± s*	58.47 ± 11.09	58.34 ± 12.14	*t* = 1.138	0.257
Male, n (%)	49 (59)	50 (70.4)	*χ^2^* = 2.991	0.084
BMI (kg/m^2^), $\overset{-}{x}$ * ± s*	25.21 ± 3.46	24.50 ± 4.21	*t* = 0.836	0.485
Smoking, n (%)	34 (37.4)	29 (40.8)	*χ^2^* = 0.851	0.356
Drinking, n (%)	26 (28.6)	27 (38)	*χ^2^* = 0.762	0.383
Diabetes mellitus, n (%)	19 (23)	23 (32.4)	*χ^2^* = 0.023	0.880
Hypertension*, *n (%)	45 (49.5)	26 (36.6)	*χ^2^* = 0.146	0.703
Hyperlipidemia, n (%)	22 (24.2)	16 (22.5)	*χ^2^* = 0.232	0.630
TC (mmol/L), $\overset{-}{x}$ * ± s*	4.34 ± 0.68	4.05 ± 0.84	*t* = 1.257	0.216
TG (mmol/L), $\overset{-}{x}$ * ± s*	1.29 ± 1.30	1.17 ± 0.48	*t* = 0.447	0.657
LDL‐C (mmol/L), $\overset{-}{x}$ * ± s*	2.21 ± 0.344	2.01 ± 0.53	*t* = −1.442	0.160
HDL‐C (mmol/L), $\overset{-}{x}$ * ± s*	1.24 ± 0.28	1.15 ± 0.30	*t* = 2.405	0.321
sdLDL‐C (mg/dL), $\overset{-}{x}$ * ± s*	34.53 ± 15.87	26.18 ± 8.04	*t* = −2.308	0.014
sdLDL‐C/LDL‐C (%), $\overset{-}{x}$ * ± s*	36 ± 15	30 ± 14	*t* = 3.052	0.002

sdLDL‐C/LDL‐C: ratio of sdLDL‐C to LDL‐C.

### 3.4 sdLDL‐C level and ACI events

Logistic regression was used to determine whether sdLDL‐C was an independent variable. The results showed that sdLDL‐C level was an independent risk factor for ACI (*P* < 0.001) (Table 5) in the basic model adjusted for age and sex (model 1) (odds ratio[OR], 1.071; 95% confidence interval [CI], 1.047‐1.097) and after additional adjustment for smoking status, alcohol consumption, BMI, diabetes mellitus, hypertension, and hyperlipidemia (model 2) (OR, 1.073; 95% confidence interval [CI], 1.048‐1.099). sdLDL‐C remained significantly associated with the risk of ACI after further adjustment for other lipid risk factors, such as LDL‐C and TC.

**Table 5:** Logistic regression analysis

	*B*‐value	SE	*Wals χ* ^2^	OR value (95% CI)	*P*‐value
Model 1^a^	0.069	0.012	33.470	1.071 (1.047‐1.097)	0.0001
Model 2^b^	0.070	0.012	33.462	1.073 (1.048‐1.099)	0.0001
Mode 3^c^	0.064	0.013	26.111	1.067 (1.041‐1.093)	0.0001

CI, confidence interval; OR, odds ratio.

a Adjusted for age and sex.

b Adjusted for model 1 variables + smoking status, alcohol consumption, body mass index, diabetes mellitus, hypertension, and hyperlipidemia.

c Adjusted for model 2 variables + total cholesterol, triglyceride, low‐density lipoprotein cholesterol, and high‐density lipoprotein cholesterol level.

### 3.5 Comparison of baseline data among ACI patients without plaques and those with stable plaques and unstable plaques

A total of 519 patients with ACI were selected. The proportion of plaque‐free patients in the three groups was 18.3%, the stable plaque group accounted for 20.8% of patients, the unstable plaque group accounted for 60.9% of patients, and the plaque groups (stable plaque group and unstable plaque group) accounted for 81.7% of the total cohort. There was no significant difference in sex, BMI, smoking status, alcohol consumption, diabetes, hypertension, hyperlipidemia, or TG between the two groups (*P* > 0.05). The age of the unstable plaque group and the age of the stable plaque group were significantly different from that of the plaque‐free group (*P < *0.05). There was no significant difference between the serum TC and LDL‐C levels in the stable plaque groups and the unstable plaque group, which were significantly higher than those in the plaque‐free group (*P < *0.05, *P < *0.001). The serum HDL‐C and sdLDL‐C levels in the unstable plaque group were higher than those in the other two groups, and the difference was statistically significant (*P < *0.05, *P < *0.001) (Table 6).

**Table 6:** Comparison of baseline data of three groups with CAS plaque stability in patients with ACI

	No plaque	Stable plaque	Unstable plaque
Number of cases, n (%)	95 (18.3)	108 (20.8)	316 (60.9)
Age (y), $\overset{-}{x}$ * ± s*	53.89 ± 10.91	62.34 ± 7.95^a^	62.84 ± 11.02^c^
Male, n (%)	63 (66.3)	77 (71.3)	200 (63.3)
BMI (kg/m^2^), $\overset{-}{x}$ * ± s*	26.70 ± 3.88	26.49 ± 5.38	26.49 ± 4.61
Smoking, n (%)	38 (40)	52 (48.1)	123 (38.9)
Drinking, n (%)	22 (23.2)	52 (48.1)	129 (40.8)
Diabetes mellitus, n (%)	9 (9.5)	24 (22.2)	67 (21.2)
Hypertension*, *n (%)	60 (63.2)	63 (58.3)	204 (64.6)
Hyperlipidemia, n (%)	67 (70.5)	74 (68.5)	196 (62)
TC (mmol/L), $\overset{-}{x}$ * ± s*	4.87 ± 1.21	5.13 ± 1.02	5.35 ± 0.99^c^
TG (mmol/L),$\overset{-}{x}$ * ± s*	1.72 ± 1.04	1.83 ± 1.08	1.86 ± 1.08
LDL‐C (mmol/L), $\overset{-}{x}$ * ± s*	3.07 ± 0.85	3.30 ± 0.78	3.54 ± 0.95
HDL‐C (mmol/L), $\overset{-}{x}$ * ± s*	1.19 ± 0.22	1.37 ± 0.27^a^	1.36 ± 0.38^d^
sdLDL‐C (mg/dL), $\overset{-}{x}$ ± *s*	26.42 ± 14.84	35.96 ± 15.60^b^	44.65 ± 18.50

a *P *< 0.05: Stable plaque group vs No plaque group.

b *P* < 0.001: Stable plaque group vs No plaque group.

c *P* < 0.05: Unstable plaque group vs No plaque group.

*P* < 0.05: Unstable plaque group vs stable plaque group.

d *P* < 0.001: Unstable plaque group vs No plaque group.

### 3.6 sdLDL‐C level and unstable plaques in CAS patients with ACI

Binary logistic regression analysis was used to determine the risk factors for unstable plaques in the ACI group. sdLDL‐C was tested as an independent with unstable plaque as a dependent variable, after adjusting for age and sex, (OR, 1.046; 95% confidence interval [CI], 1.034‐1.059) and after additional adjustment for smoking status, alcohol consumption, BMI, diabetes mellitus, hypertension, and hyperlipidemia (OR, 1.055; 95% confidence interval [CI], 1.041‐1.070). After further adjustment for other influencing factors, the analysis showed that sdLDL‐C was an independent risk factor for unstable atherosclerotic plaques (*P* < 0.001) (Table 7).

**Table 7:** Logistic regression analysis of CAS with unstable plaques in patients with ACI

	*B*‐value	SE	*Walsχ2*	OR value (95% CI)	*P*‐value
Model 1^a^	0.045	0.006	16.522	1.046 (1.034‐1.059）	0.0001
Model 2^b^	0.054	0.007	16.378	1.055 (1.041‐1.070）	0.0001
Mode 3^c^	0.051	0.007	14.281	1.053 (1.038‐1.068）	0.0001

CI, confidence interval; OR, odds ratio.

a Adjusted for age and sex.

b Adjusted for model 1 variables + smoking status, alcohol consumption, body mass index, diabetes mellitus, hypertension, and hyperlipidemia.

c Adjusted for model 2 variables + total cholesterol, triglyceride, low‐density lipoprotein cholesterol, and high‐density lipoprotein cholesterol level.

### 3.7 Correlation between CAS plaque stability and serum sdLDL‐C in patients with ACI

**Figure 2:**
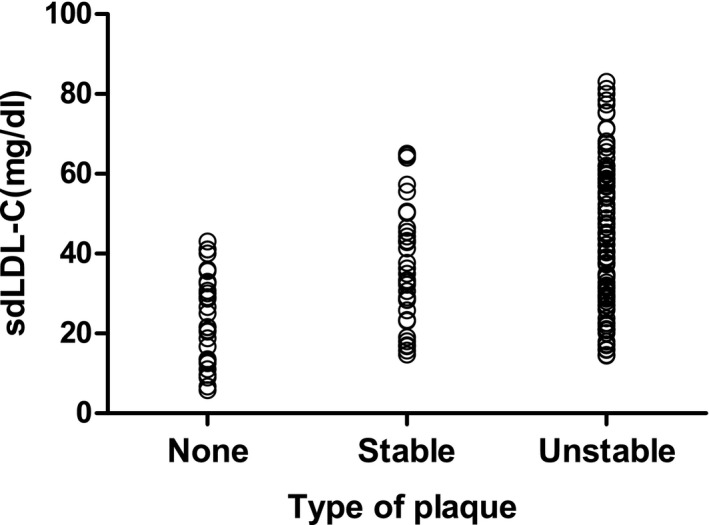
Correlation between CAS plaque stability and serum sdLDL‐C level in patients with ACI

Table 7 shows that the sdLDL‐C level gradually increases with changes in plaque properties and is an independent risk factor for unstable plaques in ACI patients. Spearman correlation analysis showed a positive correlation between sdLDL‐C levels and changes in arterial plaque properties (Table 8, Figure 2).

**Table 8:** Relationship of the stability of CAS plaque with age and sdLDL‐C level in patients with acute ischemic cerebral infarction

	*r*	*P*‐value
sdLDL‐C	0.375	0.0001

*r*, Correlation coefficient.

### 3.8 Identification of CAS with unstable plaques in patients with ACI with the receiver operating characteristic (ROC) curve of sdLDL‐C

**Figure 3:**
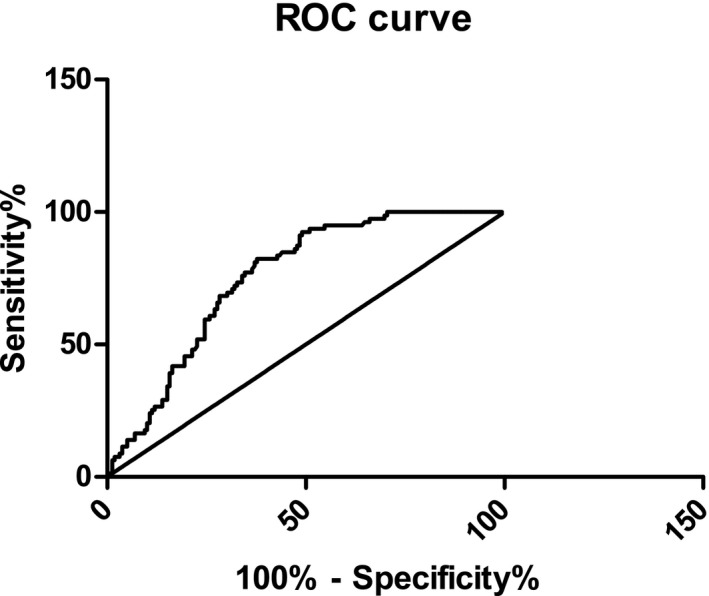
Receiver operating characteristic curves of sdLDL‐C for the identification of unstable CAS plaques in patients with ACI

With unstable plaque as the state variable and sdLDL‐C as the test variable, a ROC curve was obtained, and the area was 0.695; *P < *0.001, CI: 0.613‐0.777 (Table 9, Figure 3). The calculated Youden index has a maximum value of 0.302, a corresponding sensitivity of 63.5%, a specificity of ≈1‐0.333 = 66.7%, and a critical value of sdLDL‐C of 36.80 mg/dL.

**Table 9:** Receiver operating characteristic curves of sdLDL‐C for the identification of unstable CAS plaques in patients with ACI

	Cutoff value	Area	Sensitivity	Specificity	95% CI	
					Lower bound	Upper bound
sdLDL‐C	36.80	0.695	63.5	66.7	0.613	0.777

## 4 DISCUSSION

Cerebral infarction is the result of multiple factors. Common factors such as age, sex, hypertension, diabetes, elevated TC and LDL‐C levels, decreased HDL‐C level, smoking, and family history have prognostic value, especially LDL‐C elevation, which is considered the most important traditional risk factor for cerebral infarction. CAS is the pathological basis of ACI, and sdLDL‐C, one of the subcomponents of LDL‐C, is more potent than coronary atherosclerosis.^13^ The results of this study show that the level of sdLDL‐C in the ACI group was significantly higher than that in the control group (*P < *0.001); further studies found that the level of sdLDL‐C in the unstable plaque group was significantly higher than that in the stable plaque group and the plaque‐free group (*P < *0.05, *P* < 0.001).

Based on the mechanism by which sdLDL‐C induces atherosclerosis, it can be concluded that sdLDL‐C carries a higher risk of coronary heart disease than LDL‐C. Several large studies have evaluated the relationship between LDL‐C particle size and coronary heart disease. Sakai K et al studied sdLDL‐C levels in 345 Japanese men ≥65 years of age with stable coronary artery disease. sdLDL‐C is a more effective secondary biomarker for cardiovascular events than LDL‐C.^14^ The highest level of sdLDL‐C was reported by a project funded by the National Cardiopulmonary Hematology Institute, which had used a new automated detection method for small‐density cholesterol in the determination of coronary heart disease and atherosclerosis risk. Individuals in the higher quartile groups had a higher risk of coronary heart disease than individuals in the lowest quartile of LDL‐C levels.^15^ In addition, the National Human Genome Research Institute project and the National Institutes of Health project‐supported study, “Small, dense low‐density lipoprotein cholesterol predicts the risk of coronary heart disease based on the risk of atherosclerosis (ARIC) in community populations,” found that sdLDL‐C is significantly associated with the occurrence of coronary heart disease.^16^ To date, there have been few studies on the relationship between sdLDL‐C and ACI. The relationship between sdLDL‐C and carotid plaque stability has not yet been reported, and the pathological basis of ACI is the same as that of coronary heart disease. We considered whether sdLDL‐C can be used as an independent risk factor for ACI. The results of logistic regression analysis showed that sdLDL‐C levels were positively correlated with ACI. After adjusting for other traditional risk factors, sdLDL‐C was an independent risk factor for ACI, suggesting that sdLDL‐C may be a potential biomarker for predicting the occurrence of cerebrovascular events.

The occurrence of cerebral infarction is closely related to the stability of CAS plaques, so accurate prediction of plaque properties has become the key to preventing the occurrence of cerebral infarction.^2^, ^3^ Imaging methods can be used to judge the stability of plaque. However, traditional imaging diagnostic techniques can show only the morphology of the arterial lumen or plaque, and there is a significant lack of evaluation of plaque stability. Recent studies have found that lowering blood lipids, especially sdLDL‐C levels, can increase plaque stability,^17^ suggesting that there is an intrinsic link between sdLDL‐C levels and plaque stability. For the first time, our group used ultrasound angiography to group ACI patients according to plaque properties and analyzed the relationship between sdLDL‐C level and CAS plaques. The results showed that the incidence of CAS was 81.13% in ACI patients; the proportion of patients in the unstable plaque group (60.9%) was much larger than that in the stable plaque group (20.8%) and the plaque‐free group (18.3%), indicating that unstable plaques are closely related to the occurrence of ACI. Logistic regression was used to demonstrate that sdLDL‐C is a risk factor for unstable atherosclerotic plaques in patients with acute ischemic infarction. sdLDL‐C had a *P*‐value < 0.001 and an OR value >1, suggesting that sdLDL‐C is an important risk factor for unstable plaques. At the same time, Spearman correlation analysis showed that the level of sdLDL‐C was positively correlated with the stability of carotid plaques in patients with ACI, which was consistent with the results of logistic regression analysis. In addition, sdLDL‐C has good sensitivity and specificity for assessing CAS with unstable plaques in patients with ACI. The area under the ROC curve for sdLDL‐C was 0.695, the sensitivity and specificity were 63.5 and 66.7, respectively, and the critical value for diagnosing unstable plaques was 36.80 mg/dL.

It is traditionally thought that LDL‐C can predict risk in ACI patients, but LDL‐C levels are in the normal range in some ACI patients. The results of this study found that the level of sdLDL‐C in the high LDL‐C group of ACI patients was indeed higher than that in the normal LDL‐C group (*P < *0.05). The levels of sdLDL‐C and the ratio of sdLDL‐C to LDL‐C were significantly different between the ACI group and the control group when the level of LDL‐C is normal (*P* < 0.05). The LDL‐C level alone does not accurately reflect risk in ACI patients; even in those with normal LDL‐C levels, the risk of ACI cannot be ruled out. sdLDL‐C level and the ratio of sdLDL‐C to LDL‐C in the ACI group with normal LDL‐C levels were higher than those in the control group with normal LDL‐C levels. This result may suggest that in patients with normal LDL‐C levels, we can determine the risk of ACI by the level of sdLDL‐C and the ratio of sdLDL‐C to LDL‐C.

It has been reported in the literature that TG can regulate the particle size of sdLDL‐C.^18^ Indeed, the sdLDL‐C size was significantly larger in the high TG group than in the low TG group (*P* < 0.001), and Pearson correlation analysis showed a positive correlation between sdLDL‐C and TG, which is consistent with the findings reported by Rizzo.^18^ It has been proven that sdLDL‐C production is closely related to TG. This finding is even more meaningful considering that dyslipidemia in China is mainly attributed to hypertriglyceridemia. Furthermore, we explored the correlation between TG and CAS, and the results showed that TG is correlated with stable plaques. There was no significant difference in the grouping (*P* > 0.05), but there was a significant difference in sdLDL‐C level (*P < *0.001), which again showed that sdLDL‐C was superior to traditional factors in predicting ACI.

In summary, this study analyzed the association of sdLDL‐C level with ACI and CAS plaque stability in patients with ACI. The results showed that sdLDL‐C is not only an independent risk factor for unstable plaques but also positively correlated with CAS plaque stability. This finding indicates that the level of serum sdLDL‐C can help clinicians identify high‐risk patients so that timely prevention measures can be taken.
